# Effects of Fusarium Wilt on Cotton Cultivars with and without *Meloidogyne Incognita* Resistance in Fields

**DOI:** 10.2478/jofnem-2022-0017

**Published:** 2022-07-07

**Authors:** Terry A. Wheeler, Jessica Dotray, Cecilia Monclova-Santana

**Affiliations:** 1 Texas A&M AgriLife Research, Lubbock, TX 79403 USA; 2 Department of Plant and Soil Science, Texas Tech University, Lubbock, TX 79409 USA; 3 Texas A&M AgriLife Extension Service, Texas Tech University, Lubbock, TX 79403 USA

**Keywords:** cotton, *Fusarium oxysporum* f. sp. *vasinfectum*, Fusarium wilt, *Gossypium hirsutum*, management, *Meloidogyne incognita*, resistance

## Abstract

Cotton (*Gossypium hirsutum*) cultivar trials were conducted in four fields (6 trials total) with *Meloidogyne incognita* (Mi)/*Fusarium oxysporum* f. sp. *vasinfectum* (Fov) from 2019 to 2021. Cotton cultivars were divided into groups based on company/Mi resistance: S = susceptible to Mi; R-FM, R-DP, and R-PHY = resistance to Mi in FiberMax®, Deltapine®, and Phytogen® cultivars, respectively; ST 4946GLB2 (moderate resistance to Mi and observed field tolerance to Fov); and ST 5600B2XF (resistance to Mi). The S and R-FM groups had the highest transformed Mi densities LOG_10_(Mi + 1) (LMi = 3.22 and 3.01, respectively), while R-DP and R-PHY had the lowest LMi (2.21 and 1.85, respectively). Plant mortality (%) was higher for R-DP (28.1%) than for all other groups except ST 5600B2XF (24.8%). Mi-susceptible cultivars averaged 23.3% mortality. Relative yield (0-1 scale) was higher for ST 4946GLB2 (0.706) and R-PHY (0.635) than for R-DP (0.530), ST 5600B2XF (0.578), and S (0.491). All groups except R-DP averaged higher relative yield than the susceptible cultivars. ST 4946GLB2 had the lowest mortality (16.5%) and highest relative yield, while R-DP cultivars had the highest mortality and no difference in relative yield from the Mi-susceptible cultivars. The group of R-DP cultivars had excellent Mi resistance but were susceptible to Fov. No cultivars were identified with high resistance to Fov.

Fusarium wilt in cotton (*Gossypium hirsutum* L.) is caused by *Fusarium oxysporum* f. sp. *vasinfectum* (Fov). The disease, depending on the race of Fov, can be caused by an interaction between Fov and the southern root-knot nematode (*Meloidogyne incognita*, Mi). Atkinson (1892) first reported an association between severe wilt in cotton and nematodes. Martin et al. (1956) were the first to demonstrate the interaction in cotton by inoculating sterilized soil with no pathogen, Fov alone, Mi alone, and Fov + Mi. The interaction was demonstrated on both Fov-susceptible (“Deltapine 15”) and Fov-resistant (“Coker 100”) cultivars. As densities increased for both Fov and Mi, wilt symptoms and root damage on cotton increased (Garber et al., 1979; Starr et al., 1989; DeVay et al., 1997). It has long been realized that nematode damage in plants is density related (Seinhorst, 1965; Barker and Olthof, 1976). However, the impact that Mi has on Fusarium wilt severity is profound. Garber et al. (1979) found that 77,000 conidia/g soil were necessary to cause Fusarium wilt symptoms in the absence of Mi, but only 650 conidia/g soil were required in the presence of Mi.

A survey of Fov races in the U.S. was conducted from 2012 to 2013, and races 1, 2, 3, 4, and 8 were detected (Cianchetta et al., 2015). In the survey, of the 24 isolates collected in Texas (collected primarily in the Southern High Plains), 18 isolates were identified as race 1, 5 isolates as race 2, and 1 isolate as race 3. Since that survey, race 4 has been found in Texas in the far west counties of El Paso and Hudspeth (Halpern et al., 2018). Fov races 1 and 2, when combined with Mi, are associated with the destructive interaction on cotton (Cianchetta and Davis, 2015), resulting in plant mortality, stunting, chlorotic leaf symptoms, vascular and root necrosis, and substantial yield loss.

Management of the Fov/Mi complex has been more challenging from the fungal side, and hence most successful options involve reduction of Mi density through nematicides or host resistance to Mi. A high level of resistance to Mi was found by crossing “Coker Clevewilt 6” to “Mexico Wild” (PI563649) resulting in “Auburn 623RNR” (Shepherd, 1974). An important tool in the development of commercial cultivars with Mi resistance occurred when SSR markers were identified that were associated with the two Mi-resistant genes in Auburn 623RNR (Gutierrez et al., 2010). Marker assisted selection using CIR 316-201 on chromosome 11 and BNL 3661-185 on chromosome 14 was verified by recreating the original crosses between “Coker Clevewilt 6” and “Mexico Wild”, and rapidly selecting even more resistant lines (Jenkins et al., 2012). Commercial cultivars with high levels of resistance to Mi and good yielding ability (average of 17% yield increase compared to susceptible cultivars) can reduce nematode densities by >90% compared with susceptible cultivars (Wheeler et al., 2020). Other methods to reduce the density of Mi can include crop rotation to non-hosts such as peanuts or the use of nematicides. The nematicide aldicarb can reduce galling caused by Mi, reduce the incidence of Fusarium wilt, and increase yield in Fov/Mi infested fields (Colyer et al., 1997; Wheeler and Gannaway, 2005). The use of fumigants at high rates in a Fov/Mi cotton field increased yield by 200% to 400%, and decreased wilt related mortality, frequency of infection, vascular discoloration, and root-knot nematode galling (Jorgenson et al., 1978). Fumigation only increased cotton yields for cultivars that were susceptible to the Fov/Mi complex or only partially resistant, while Auburn 623RNR had similar yield both with and without fumigation (Shepherd, 1982).

Crop rotation has not been successful in reducing Fov to a level where it is no longer a threat to cotton (Davis et al., 2006). The fungus can persist for long periods in the soil in the form of chlamydospores, and even if the fungal populations are reduced, they can build up again rapidly once a susceptible crop is planted (Smith, 2007). The fungus can also be recovered from senescing plant tissue due to its saprophytic abilities (Davis et al., 2006).

The earliest selections for Fov resistance were identified from *Gossypium barbadense* Sea Island Pima ‘Rivers’ (Orton, 1907). It was determined that the inheritance of near immunity to wilt in Sea Island cotton was due to two dominant factors (Smith, 1953). This source of strong resistance was with regard to Fov race 1. Upland (*G. hirsutum*) cotton was thought to have a single dominant factor and did not possess cultivars with as much resistance as Sea Island Pima cultivars. “Jackson’s Limbless” was selected from a founder germplasm (“Burling’s Mexican”) for American upland cotton, with good resistance to Fusarium wilt, and “Dillon” was developed from it in 1905 (Orton, 1908). “Dixie” was then also developed from that source and when crossed with “Triumph” (“Dixie-Triumph”) was a successful cultivar combining wilt tolerance with better agronomics (Smith, 1953). An early *G. hirsutum* line, developed from founder germplasm ‘Mexican’ or ‘Petit Gulf’ with resistance to both Fov and Mi was ‘Cook 307-6’ (in 1915) and ‘Coker Clevewilt’ (in 1931) (Wilhelm, 1981; Zhang et al. 2015). These two cultivars were heavily utilized in development of Fusarium wilt tolerant cultivars in the U.S. “Auburn 56” was derived from “Cook 307-6” × “Coker 100” and had resistance to both Fov and Mi. “Coker Clevewilt” was involved with the development of LA 887 (plant variety protection PVP 009100065), which was utilized in the development of Mi partially resistant Stoneville varieties such as “ST 5599BR” (PVP 200300279), “ST 5458B2RF” (PVP 200800229), and “ST 4946GLB2” (PVP 201300350, crossed through the intermediary “ST 457” PVP 20020027, which was developed through a cross with ST LA 887).

In the Southern High Plains of Texas, Fov/Mi complex is present in many cotton fields, though it is not as common as the 500,000 ha to 700,000 ha that are infested with Mi (Starr et al., 1993; Wheeler et al., 2000). In 2003, there were some cotton fields with severe Fusarium wilt in this region, where >50% mortality occurred (T. Wheeler, personal observations). There was a consistent theme in these fields, that the newly introduced conventional FiberMax® cultivars had been planted for 2 to 3 consecutive years. After 2014, severe Fusarium wilt occurred with producers planting some new cultivars with excellent resistance to Mi. In both situations, small plot variety trials were performed to determine if certain company’s cultivars were more susceptible to the Fov/Mi complex. In 2003, the question was asked whether the conventional FiberMax cultivars were more susceptible than other Mi-susceptible cultivars, and cultivars with partial resistance to *M. incognita*. In the situation that developed after 2014, the question was asked whether sources of Mi resistance from different companies might differ in susceptibility to Fusarium wilt. The objective of this project was to evaluate different cotton cultivars (grouped by company) with and without Mi resistance for response to Fov.

## Materials and Methods

Small plot replicated tests with commercial cotton cultivars were planted in a producer field at one location in Dawson County (Table 1) in 2004 and 2005, where severe Fusarium wilt had developed in 2003. Small plot replicated tests were also planted in three producer sites (Table 1) in 2019 (Gaines, Hall, and Lynn Counties); two sites in 2020 located in Cochran and Hall Counties; and one site (Hall County) in 2021. Each test had between 24 and 48 entries, with four replications per entry, arranged in a randomized complete block design. Plots were two rows wide (1 m centers) and 10.67 m long. A list of Mi-resistant entries in the tests can be found in Table 2. Plots were irrigated with a center pivot system at all locations, though irrigation capacities and yields differed greatly between sites. All sites were naturally infested with Mi and Fov race(s) 1 and/or 2 in the 2019 to 2021 tests. No race determination was made with the Dawson County test site. Fertilizer, irrigation, and other practices were dictated by the producer’s normal management.

**Table 1:** List of test locations, dates of planting, and other field specific information.

					Dates^z^ for field activities			
		Latitude/	Soil			Initial	Final	Nematode
Year	County	Longitude	Series	Planting	Harvest	Stand	Stand	Sampling
2020	Cochran	33.65256 −102.6565	Amarillo fine sandy loam	5/19	11/10	6/11	11/10	8/10
2004	Dawson	32.78918	Patricia loamy	5/6	12/3	6/3	8/28	8/30
2005	Dawson	−102.0631	fine sand	5/14	11/11	6/7	7/20	…
2019	Gaines	32.73526	Patricia	5/17	11/15	6/12	11/15	10/7
		−102.8783	fine sand					
2019	Hall	34.36166	Miles loamy fine	5/29	11/4	6/11	11/4	9/3
2020	Hall	−100.9165	sand	5/15	11/16	6/4	11/16	8/17
2021	Hall			5/10	11/9	5/24	11/9	8/18
2019	Lynn	32.91140	Amarillo fine	5/15	11/14	6/6	11/14	8/20
		−102.0075	sandy loam					

z Dates are formatted with month/day.

**Table 2:** List of cotton cultivars used in the trials with tolerance or resistance to Mi.

		Plant Variety	
Company	Cultivar	Certificate^z^	Company description of Resistance
BASF	FM 1621GL	201900404	4 on 1-5 scale, 5 = resistant, Mi tolerant
BASF	FM 1730GLTP		Mi/Fusarium wilt tolerance: very good (Anonymous, 2021)
BASF	FM 1911GLT	201600407	4 on 1-5 scale, 5 = resistant, Mi tolerant
Stoneville	LA 887	009100065	Mi Resistant
BCS	ST 5599BR	200300279	Mi Moderately resistant
BASF	ST 4946GLB2	201300350	4 on 1-5 scale, 5 = resistant, Mi tolerant
BASF	ST 5600B2XF		Mi resistance (Anonymous, 2021)
BCS	DP 1747NR B2XF	201700046	4 on a 1-4 scale, 4 = Mi resistant
BCS	DP 1823NR B2XF		Mi resistant; Albers and Gholston (2018)
BCS	DP 2141NR B3XF		Mi resistant; Albers et al. (2021)
BCS	DP 2143NR B3XF		Mi resistant; Albers et al. (2021)
Corteva	PHY 320 W3FE		2-gene resistance to Mi (Anonymous, 2021)
Corteva	PHY 332 W3FE	202000220	Resistance to Mi
Corteva	PHY 350 W3FE		Highly Mi resistant (Anonymous, 2021)
Corteva	PHY 394 W3FE		Resistance to Mi (Anonymous, 2021)
Corteva	PHY 400 W3FE		Resistance to Mi (Anonymous, 2021)
Corteva	PHY 411 W3FE		Resistance to Mi (Anonymous, 2021)
Corteva	PHY 443 W3FE	202000221	Resistance to Mi
Corteva	PHY 480 W3FE		Resistance to Mi (Anonymous, 2021)
Corteva	PHY 500 W3FE		Resistance to Mi (Anonymous, 2021)
Corteva	PHY 545 W3FE		Resistance to Mi (Anonymous, 2021)
Corteva	PHY 580 W3FE		Resistance to Mi (Anonymous, 2021)

z Description of Mi resistance is based on the plant variety protection certificate. Mi, *Meloidogyne incognita*.

Data collected (Table 1 for dates) included plant stand on either one or both rows once plants had emerged, but before Fusarium wilt symptoms began, and plant stand at harvest, or in the case of the 2004 to 2005 trials, once stands were stable (plants had stopped dying) in July or August. Plots were soil sampled in August or later (Table 1) to assay for root-knot nematode. Samples consisted of six cores per plot collected with a narrow-bladed (40 cm depth, 15 cm width at top, and 8 cm width at the bottom) shovel to a depth of 20 cm, close to the taproot. The top 6 cm of soil was discarded and then soil from 6 cm to 20 cm depth, including some roots, was removed. The soil was mixed in a bucket and then a subsample of 1,000 cm^3^ soil was removed and placed in a plastic bag. The soil samples were refrigerated for <2 weeks before being assayed for root-knot nematode second-stage juveniles (J2) and eggs. The test in 2005 was not sampled for nematodes.

A pie-pan assay with 200 cm^3^ soil + root fragments was used to extract J2 over 48 hr (Thistlethwayte, 1970). The circular pie-pans were made of glass and wire mesh (0.64 cm diameter) was placed in the pie-pan. Two pieces of facial tissue (2-ply) were laid on top of the mesh and then the soil sample was placed on the facial tissue. Tap water (250 ml) was gently added to the pie-pan without disturbing the soil, and then the wet facial tissues were arranged around the soil to hold it out of the water. A cover was placed over the pie-pan to eliminate evaporation. The extracted J2 were enumerated by concentrating the extracted liquid to 100 ml and then counting a 5 ml aliquot. A second assay with 500 cm^3^ soil was used to extract root-knot nematode eggs. The soil + root fragments were placed in a bucket with water (combined volume 3 l of water + soil) and stirred for 10 s. After allowing to settle for 15 s, the contents were poured over a sieve with a pore size of 230 μm and the root fragments caught on the sieve were washed into a beaker in 100 ml tap water and mixed on a stir plate for 5 min in NaOCl (0.525%) (Hussey and Barker, 1973). The mixture was poured through a sieve with a pore size of 230 μm, stacked over a sieve with a pore size of 25 μm. The contents from the bottom sieve were rinsed with tap water, washed into a beaker, dyed with acid fuchsin (Byrd et al., 1983), and the eggs were enumerated from a 5 ml aliquot taken out of the 150 ml total volume.

Plants outside of the test area which exhibited signs of Fusarium wilt were collected and *Fusarium* was isolated from the vascular system. The isolates were single-spored and stored until species and race typing could be performed. DNA extraction from mycelia was performed using Zymo Quick DNA Fungal/Bacterial Miniprep kit (Zymo Research Corp; Irvine, CA). DNA was used to run PCR using four genetic regions, translation elongation factor (EF-1_α_), phosphate permease-like protein (PHO), β-tubulin (BT), and intergenic spacer region (IGS). Primer sequences and thermocycler setting followed Cianchetta et al. (2015). PCR products were sequenced using Sanger Sequencing Platform at Molecular Cloning Laboratories. Bioinformatics used GenBank references to identify the isolates using the references used by Cianchetta et al. (2015). MEGA X software (https://www.megasoftware.net/pdfs/kumar_stecher_2018.pdf) was used for alignment and phylogenic analysis using the MUSCLE algorithm interphase (Tamura et al., 2013). For phylogenic analysis, we used neighbor-joining with 2,000 bootstraps, using all sequences in a concatenated approach (Cianchetta et al., 2015).

The plots were mechanically harvested with a cotton stripper designed to weigh the plot yield on load cells. Stripper plot yields consist of lint, seed, and plant debris. A 1,000 g sample was collected from harvested plots and two replications were ginned from each entry to determine lint percentage of the harvest weights.

Plant mortality was calculated as: ((Initial stand – final stand)/Initial stand) × 100. *M. incognita* density (Mi) was calculated by the number of egg/500 cm^3^ soil + (2.5 × number of J2/200 cm^3^ soil). A transformation of Mi density, LMi = LOG_10_(Mi + 1) was used for analysis. Yields were adjusted to relative yield on a 0 to 1 scale, so that all six trials from 2019 to 2021 could be combined for analysis. Relative yield = (plot lint yield – MinLY)/(MaxLY – MinLY); where MinLY = lint yield (LY) for the plot with the lowest lint yield at a location, and MaxLY = yield for the plot with the highest lint yield at a location. Cultivar was assigned to a category based primarily on its Mi resistance (based on company description) and company origin. The categories for the period 2004 to 2005 were FM-conventional cultivars; FM-transgenic cultivars; Mi partially resistant cultivars; and all other cultivars. In the period 2019 to 2021, the categories were: Mi susceptible (S); Mi-resistant FiberMax (R-FM); “ST 4946GLB2”; “ST 5600B2XF”; Mi-resistant Deltapine (R-DP); and Mi-resistant Phytogen (R-PHY). Groups of cultivars (based on Mi resistance by company) were used in the analyses rather than individual cultivars because Mi-resistant cultivars are often developed from the same source(s) within a company. ST 4946GLB2 was the Fov and partially Mi-resistant check and was included at all tests (2019–2021). ST 5600B2XF, which was not bred by Stoneville® Cotton, and has an unknown lineage, was also in a separate Mi-resistant category.

The tests in 2004 and 2005 contained two variables, cultivar and aldicarb, in a factorial arrangement. All plots that received aldicarb were eliminated from the analysis. The individual cultivar means from 2004 were presented previously (Wheeler and Gannaway, 2005). The Mi density in Hall Co. in 2019 in replication 4 averaged 9 Mi/500 cm^3^ soil, and thus that replication was deleted from the data set. All other site-years and replications had sufficient Mi density (average >800/500 cm^3^ soil) to be utilized.

The various groups were analyzed for percentage mortality, LMi, and lint yield within each test site using mixed model analyses (PROC GLIMMIX, SAS version 9.4; SAS Institute, Cary, NC), where group was the fixed variable and replication, or year and replication were the random variables. Significant differences between categories were determined by *t*-tests, at *P* < 0.05. Pearson’s correlation coefficient (PROC CORR) was determined for mortality, Mi density, LMi, and lint yield at each site. Significant relationships between mortality and LMi, the quadratic terms, and the interaction term (mortality × LMi), to describe lint yield, was determined for each site using PROC STEPWISE. For a model to be acceptable, all variables were significant at *P* < 0.05, and then the highest *R*^2^ value determined the selected model. This procedure also provided the partial *R*^2^ for each accepted variable. For data sets from the period 2019 to 2021, all data were combined, and analyzed using a mixed model analysis for percentage mortality, LMi, and relative yield. The fixed variable was group, and the random variables were year, site, and replication.

## Results

**Figure 1:**
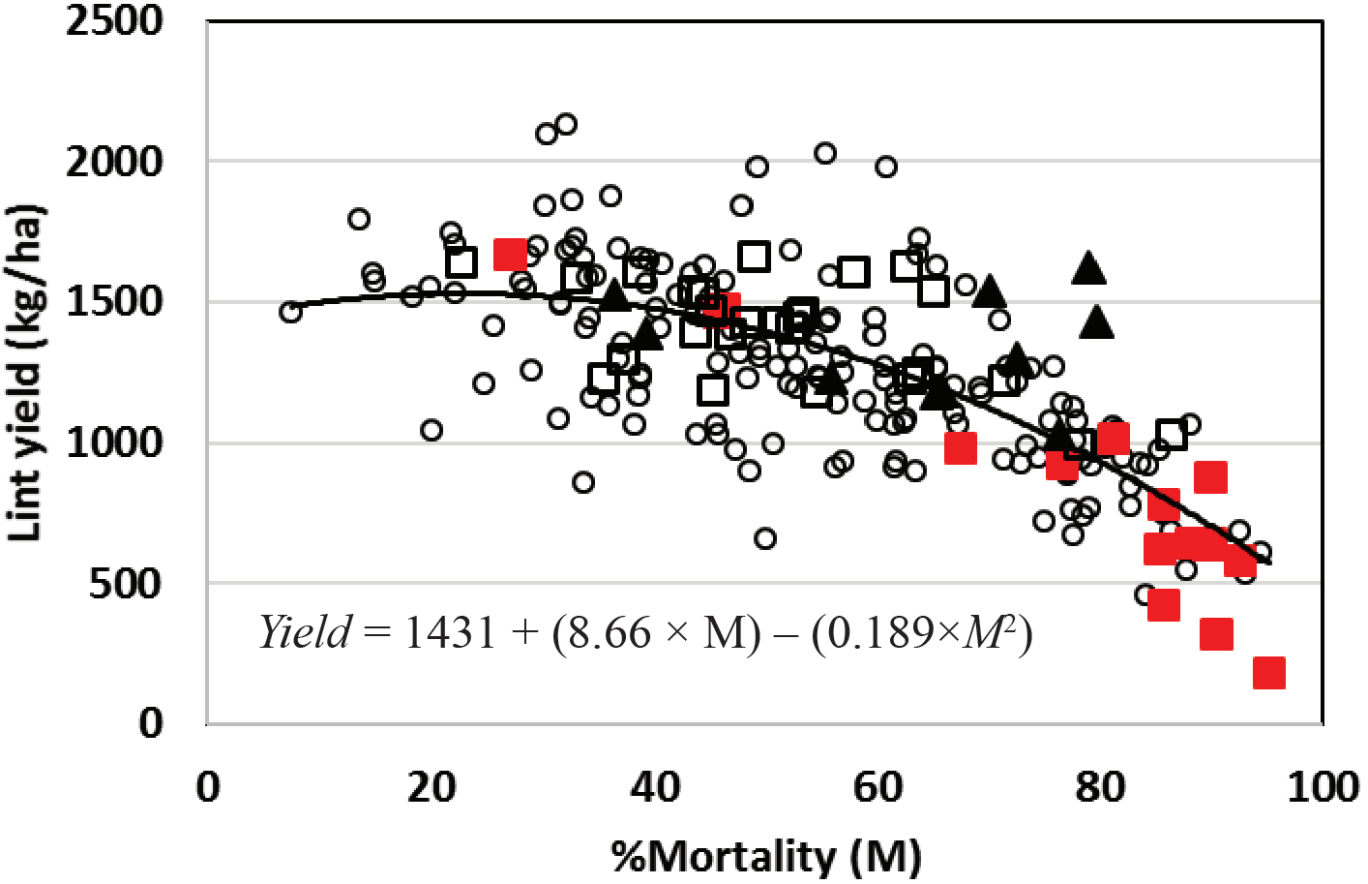
The effect of plant mortality (M) caused by the Fusarium wilt/Meloidogyne incognita (Mi) complex on cotton cultivars grouped by susceptibility to Mi. All susceptible cultivars to Mi, with the exception of Fibermax® (FM) cultivars ◯; FM-conventional cultivars ■; FM-transgenic cultivars ◻; Mi-resistant cultivars ▲. A quadratic model based on plant mortality was fitted to yield.

In the trials conducted during 2004 and 2005, the conventional FM group (FM 819, FM 832, FM 958, and FM 966) had higher mortality (69.2%) than the transgenic FM group (58.7%), or Mi-susceptible group (55.7%) (Table 3). The Mi-resistant cultivars (ST LA887 and ST 5599BR) were intermediate (60.3% mortality) and not different from any of the groups. Transformed *M. incognita* density (LMi) was lower for the resistant group (1.93) than for all other groups (2.90–3.16, Table 3). Lint yield was higher for the Mi-resistant group (1,448 kg/ha) than for the Mi-susceptible group (1,252 kg/ha) and conventional FM group (941 kg/ha) (Table 2). FM-transgenic group lint yield (1,309 kg/ha) was not different from the yields corresponding to Mi-susceptible or Mi-resistant groups. The conventional FM group had lower yields than any other group. Lint yield was correlated with percentage mortality (*r* = −0.699, *P* = 0.0001), but not with Mi or LMi. Lint yield (kg/ha) was best fitted with a quadratic model using percentage mortality (Fig. 1; Eq. 1).

**Table 3:** **Effect of cultivar groups that include FiberMax**®**, other Mi-susceptible cultivars, and Mi-resistant cultivars on Fusarium wilt mortality, Mi density,a nd yield**.

				Lint
Cultivar	%	Mi/500		Yield
Group^z^	Mortality	cm^3^ soil	LMi^y^	(kg/ha)
FM-conventional	69.2 a^x^	1,905	2.90 a	941 c
FM-transgenic	58.7 b	1,507	3.16 a	1,309 ab
Normal	55.7 b	1,742	3.02 a	1,252 b
Mi resistant	60.3 ab	607	1.93 b	1,448 a
Prob>F	0.001	0.438	0.011	0.001

z FM-conventional group contained FM 819, FM 832, FM 958, and FM 966; FM-transgenic group contained FM 960B2R, FM 960BR, FM 960RR, FM 966LL, FM 981LL, FM 989BR, and FM 989RR; the non-FiberMax, susceptible group contained *M*. *incognita* susceptible cultivars, which can be found in Appendix 1. Mi-resistant cultivars were ST LA887 and ST 5599 BR. ^y^LMi was LOG_10_(*M*. *incognita* (eggs + second-stage juveniles)/500 cm^3^ soil + 1). ^x^Values represent the LS means from tests conducted in 2004 and 2005 using a mixed model analysis. LS means with the same letter are not significantly different (*P =* 0.05). LS, least square; Mi, *Meloidogyne incognita*.

(1) *Yield* = 1431 + (8.86 × *M*) − (0.189 × *M*^2^), where M = % mortality, *P* = 0.0001, *R*^2^ = 0.54.

With the data sets from 2019 to 2021, percentage mortality was significantly affected by group only at the Hall Co. site (Table 4). At that site, percentage mortality was higher for R-DP (22.9%) than for all other groups (8.5%–14.9%) except ST 5600B2XF (20.3%). While the other sites did not have significant group differences, the R-DP group numerically had the highest mortality at Cochran and Lynn Counties, though the susceptible group had the highest mortality at the Gaines County site. When percentage mortality was analyzed across all six data sets, R-DP group had higher mortality (28.1%) than all other groups except for ST 5600B2XF (24.8%) (Table 5). The Mi-susceptible group had 23.3% mortality, and ST 4946GLB2 numerically had the lowest mortality at 16.5%.

**Table 4:** Effect of cultivars with resistance to Mi and Mi-susceptible cultivars on plant mortality (%) caused by *Fusarium oxysporum* f. sp. *vasinfectum* races 1 and 2.

Cultivar	County location of tests			
Group^z^	Cochran	Gaines	Hall	Lynn
Susceptible	13.5	43.3	14.9 bc^y^	20.4
R-FM	13.2	…	14.4 bc	7.0
ST 4946GLB2	4.7	37.5	8.5 c	13.6
ST 5600B2XF	14.3	35.4	20.3 ab	24.0
R-DP	16.7	42.8	22.9 a	26.0
R-PHY	11.9	…	13.3 bc	24.1
Prob>F	0.732	0.701	0.013	0.317

z An entire list of cultivars can be found in Appendix 1. R-FM were Mi-resistant FM 1621GL, FM 1730GLTP, and FM 1911GLT; R-DP were Mi-resistant DP 1747NR B2XF, DP 1823NR B2XF, DP 2141NR B3XF, and DP 2143NR B3XF; R-PHY were Mi-resistant PHY 320 W3FE, PHY 350 W3FE, PHY 400 W3FE, PHY 480 W3FE, PHY 500 W3FE, PHY 545 W3FE, and PHY 580 W3FE. ^y^Values represent the LS means from tests conducted from 2019 to 2021 using a mixed model analysis. LS means with the same letter are not significantly different (*P =* 0.05). LS, least square; Mi, *Meloidogyne incognita*.

**Table 5:** Effect of Mi resistance/ tolerance by different companies and Mi-susceptible cultivars on Mi density, mortality (%) by *Fusarium oxysporum* f. sp. *vasinfectum* races 1 and 2, and relative yield from six trials.

		%	Relative	
Category^z^	LMi^y^	Mortality	Yield^x^	N
Susceptible	3.22 a^w^	23.3 b	0.491 d	378
R-FM	3.01 a	21.0 b	0.600 bc	37
ST 4946GLB2	2.78 ab	16.5 b	0.706 a	23
ST 5600B2XF	2.33 bc	24.8 ab	0.578 bc	20
R-DP	2.21 c	28.1 a	0.530 cd	46
R-PHY	1.85 c	21.4 b	0.635 ab	89
Prob>F	0.0001	0.011	0.0001	

z An entire list of cultivars can be found in Appendix 1. R-FM were Mi-resistant FM 1621GL, FM 1730GLTP, and FM 1911GLT; R-DP were Mi-resistant DP 1747NR B2XF, DP 1823NR B2XF, DP 2141NR B3XF, and DP 2143NR B3XF; R-PHY were Mi-resistant PHY 320 W3FE, PHY 350 W3FE, PHY 400 W3FE, PHY 480 W3FE, PHY 500 W3FE, PHY 545 W3FE, and PHY 580 W3FE. ^y^LMi = LOG_10_(Mi/500 cm^3^ soil + 1). ^x^Relative yield = (plot yield – minimum plot yield in the test)/(maximum plot yield in the test – minimum plot yield in the test). ^w^Values represent the LS means from tests conducted in 2019 to 2021 using a mixed model analysis. LS means with the same letter are not significantly different (*P =* 0.05). LS, least square; Mi, *Meloidogyne incognita*.

LMi was significantly affected by cultivar group for all locations (Table 6). The R-PHY group had significantly lower LMi than all other groups in Lynn County, and numerically the lowest density in Hall County. This group was not planted at the Gaines County site, due to a request from the producer to limit non-dicamba tolerant cultivars to one entry (ST 4946GLB2). R-DP had the lowest LMi at Cochran County and significantly lower LMi than the S group at Gaines and Hall Counties. When analyzed across all six trials, LMi was higher for the susceptible group (LMi = 3.22) and R-FM (3.01) than for R-PHY (1.85), R-DP (2.21), and ST 5600B2XF (2.33) (Table 5). ST 4946GLB2 had higher LMi (2.78) than R-DP and R-PHY.

**Table 6:** Effect of cultivars with resistance to Mi and Mi-susceptible cultivars on Mi density.

			County location of tests					
Cultivar	Cochran		Gaines		Hall		Lynn	
Group^z^	Mi	LMi^x^	Mi	LMi	Mi	LMi	Mi	LMi
Susceptible	9,070	3.61 a^y^	3,789	3.11 a	4,444	2.93 a	5,406	3.35 a
R-FM	3,952	3.35 ab	…	…	2,785	2.68 ab	4,510	3.56 a
ST 4946GLB2	2,640	3.02 ab	1,145	2.96 ab	1,127	2.42 ab	1,390	2.95 ab
ST 5600B2XF	1,050	2.90 b	370	1.96 b	740	2.13 abc	1,110	2.33 b
R-DP	763	1.85 c	693	2.13 b	1,421	1.95 bc	785	2.76 ab
R-PHY	2,999	3.26 ab	…	…	466	1.51 c	165	1.26 c
Prob>F	0.001	0.001	0.096	0.003	0.001	0.001	0.321	0.001

z The entire list of cultivars can be found in Appendix 1. R-FM were Mi-resistant FM 1621GL, FM 1730GLTP, and FM 1911GLT; R-DP were Mi-resistant DP 1747NR B2XF, DP 1823NR B2XF, DP 2141NR B3XF, and DP 2143NR B3XF; R-PHY were Mi-resistant PHY 320 W3FE, PHY 350 W3FE, PHY 400 W3FE, PHY 480 W3FE, PHY 500 W3FE, PHY 545 W3FE, and PHY 580 W3FE. ^y^Values represent the LS means from tests conducted from 2019 to 2021 using a mixed model analysis. LS means with the same letter are not significantly different (*P =* 0.05). ^x^LMi was LOG_10_(*M*. *incognita* (eggs + second-stage juveniles)/500 cm^3^ soil + 1). LS mean separations were only performed on LMi, not on Mi. LS, least square; Mi, *Meloidogyne incognita*.

Lint yield was significantly affected by group at all locations (Table 7). At the Cochran County site, R-FM, R-PHY, and ST 4946GLB2 had higher yields than the susceptible cultivars or R-DP. At the Gaines County site, ST 4946GLB2 had higher yields than ST 5600B2XF and susceptible cultivars. At the Hall County site, the susceptible cultivars had lower yields than all other groups except for R-DP. At the Lynn County site, susceptible cultivars yielded less than R-FM, ST 4946GLB2, and R-PHY. Relative yield was highest for ST 4946GLB2 (0.7058), and significantly higher than all groups except R-PHY (0.6351, *P* = 0.112) (Table 5). The relative yield for R-DP (0.5303) did not differ from the relative yield for the susceptible cultivars (0.4910). Relative yield for R-FM (0.600) and ST 5600B2XF (0.5782) were intermediate and significantly different from both ST 4946GLB2 and susceptible cultivars.

**Table 7:** Effect of cultivars with resistance to Mi and Mi-susceptible cultivars on lint yield in fields with *Fusarium oxysporum* f. sp. *vasinfectum* races 1 and 2.

Cultivar	County locations of tests			
Group^z^	Cochran	Gaines	Hall	Lynn
Susceptible	840 bc^y^	216 b	1,696 b	732 b
R-FM	954 a	…	1,846 a	864 a
ST 4946GLB2	1,038 a	376 a	2,026 a	877 a
ST 5600B2XF	952 ab	208 b	2,006 a	761 ab
R-DP	770 c	268 ab	1,824 ab	837 ab
R-PHY	1,004 a	…	1,970 a	847 a
Prob>F	0.001	0.007	0.001	0.008

z An entire list of cultivars can be found in Appendix 1. R-FM were Mi-resistant FM 1621GL, FM 1730GLTP, and FM 1911GLT; R-DP were Mi-resistant DP 1747NR B2XF, DP 1823NR B2XF, DP 2141NR B3XF, and DP 2143NR B3XF; R-PHY were Mi-resistant PHY 320 W3FE, PHY 350 W3FE, PHY 400 W3FE, PHY 480 W3FE, PHY 500 W3FE, PHY 545 W3FE, and PHY 580 W3FE. ^y^Values represent the LS means from tests conducted from 2019 to 2021 using a mixed model analysis. LS means with the same letter are not significantly different (*P* = 0.05). LS, least square; Mi, *Meloidogyne incognita*.

In Cochran County, lint yield was negatively correlated with mortality (*r* = −0.24, *P* = 0.003). Mortality was positively correlated with Mi (*r* = 0.23, *P* = 0.004) and LMi (*r* = 0.20, *P* = 0.015). At Gaines County, lint yield was negatively correlated with mortality (*r* = −0.68, *P* = 0.0001), Mi (*r* = −0.26, *P* = 0.010), and LMi (*r* = −0.38, *P* = 0.001), and mortality was correlated with Mi (*r* = 0.45, *P* = 0.0001). In Hall County, lint yield was negatively correlated with Mi (*r* = −0.21, *P* = 0.001) and LMi (*r* = −0.16, *P* = 0.009). In Lynn County, lint yield was negatively correlated with LMi (*r* = −0.27, *P* = 0.0001).

**Figure 2:**
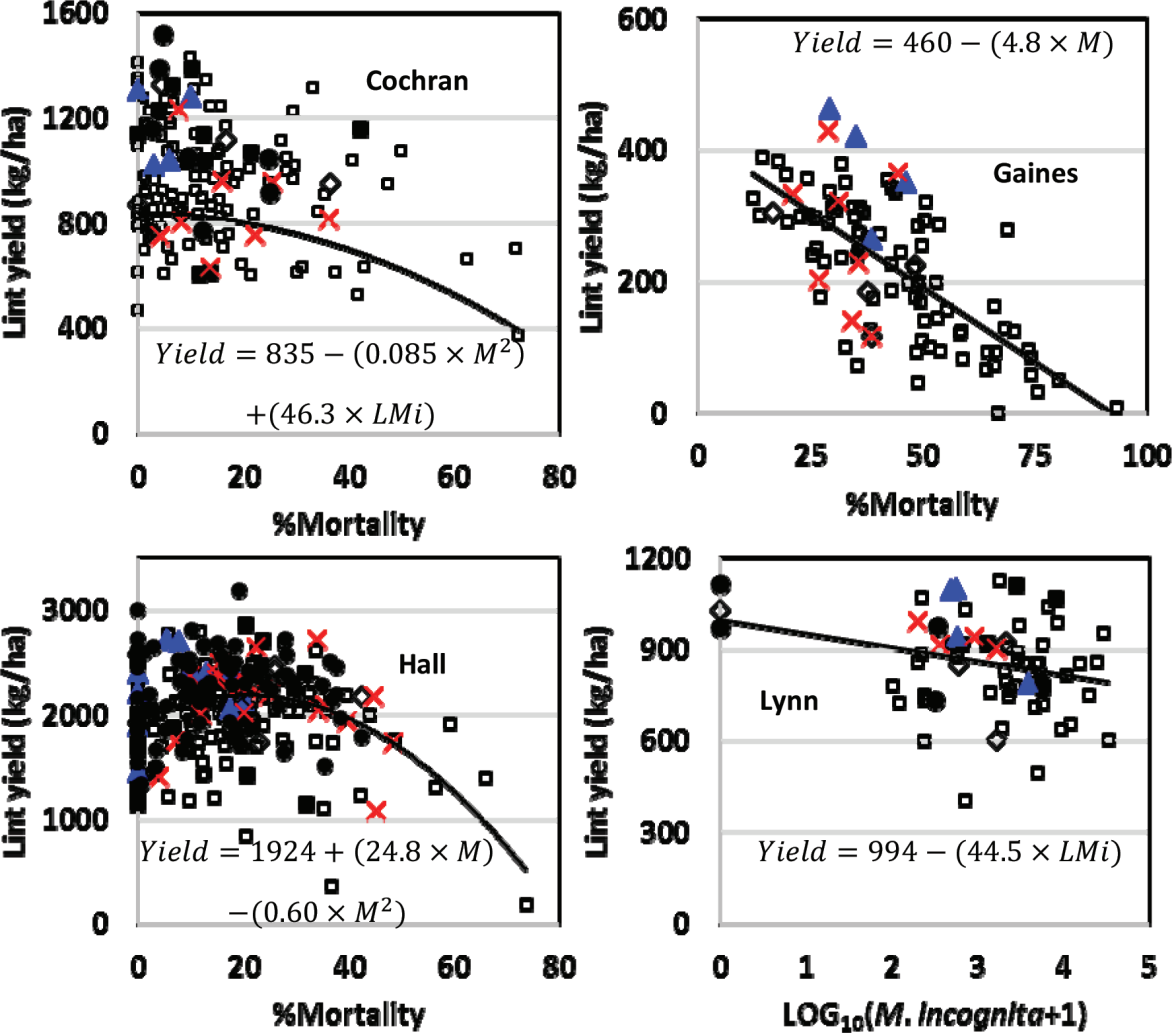
Models were fitted to lint yield collected from Fusarium wilt/Mi disease complex fields in four counties (six trials total). The best fitting factor [percentage mortality (M), transformed Mi (LMi)] was fitted to each location. Mi-susceptible ◻, Mi-resistant Fibermax ◼, ST 4946GLB2 ▲, ST 5600B2XF ◊, Mi-resistant Deltapine ×, and Mi-resistant Phytogen ● are shown for each County trial(s). A list of Mi-resistant cultivars is found in Table 2. A list of all cultivars in each trial is in Appendix 1. Mi, *Meloidogyne incognita*.

In Cochran County, lint yield (kg/ha) was fitted to a quadratic term for percentage mortality and a linear term with LMi (Fig. 2; Eq. 2), mortality^2^ had a partial *R*^2^ = 0.072, and LMi had a partial *R*^2^ = 0.033.

2) *Yield* = 835 − (0.0847 × *M*^2^) + (46.3 × *LM*i ), *P* = 0.0001, *R*^2^ = 0.11.

In Gaines County, Lint yield (kg/ha) was fitted to percentage mortality (Fig. 2; Eq. 3):

3) *Yield* = 460 − (4.82 × *M*), *P* = 0.0001, *R*^2^ = 0.45.

Fusarium wilt was most severe in this Gaines County test, and even though the irrigation was terminated prematurely (presumably due to severe disease and subsequent low yield potential), the percentage mortality explained three to six times more of the variation in yield than for the other tests.

In Hall County, Lint yield (kg/ha) was fitted to a quadratic model with percentage mortality (Fig. 2; Eq. 4):

4) *Yield* = 1924 + (24.8 × *M*) − (0.596 × *M*^2)^, *P* = 0.0001, *R*^2^ = 0.15.

In Lynn County, Lint yield (kg/ha) was described only by LMi (Fig. 2; Eq. 5)

5) *Yield* = 994 - (44.5 × L*M*i ), *P* = 0.025, *R*^2^ = 0.07.

Two races of Fov were identified across all locations from 2019 to 2021. In 2019, the Gaines County site isolate was molecularly characterized as Fov race 2, while Lynn and Hall County sites were Fov race 1. In 2020, races 1 and 2 both occurred in the same field at the Cochran County site. For all 3 yr, only race 1 was found at Hall County.

## Discussion

Tolerance and/or resistance to Fusarium wilt (referring only to those races that require *M. incognita* assistance) has been improved in cotton for many years (Kappelman, 1980, 1982; Zhang et al., 2015). The severe Fusarium wilt problem that occurred in 2003 appeared to be the result of multiple years of planting conventional FiberMax cultivars. Conventional FiberMax cultivars, which were developed in Australia, were introduced into the U.S. around 1999 (Anonymous, 1999). The first observed cases of Fusarium wilt in Australia occurred in 1993 (Kochman, 1995) and isolates of Fov were identified as something unique to Australia and were not races 1 and 2 (Davis et al., 1996). This would mean there was no selection pressure by Fov races 1 and 2 on the germplasm used in developing the conventional FiberMax cultivars grown in the U.S. While these cultivars may have been highly susceptible to Fov, the unusually high mortality for all tested cultivars in 2004 and 2005 suggest that these conventional FiberMax cultivars were also responsible for increasing soil densities of Fov and Mi to levels higher than normal. Fusarium wilt severity is a function of both Mi and Fov inoculum density (Garber et al., 1979; Starr et al., 1989; DeVay et al., 1997). Chawla (2011) planted the Mi-susceptible “FM 9058F” (PVP 200700206) and “ST 4554B2RF” (PVP 200700046) in microplots and sampled the soil for Fov densities for 3 yr. The soil densities of Fov were similar at the start of the experiment (4.6 and 4.4 colony forming units (CFU) × 105/cm^3^ soil for FM 9058F and ST 4554B2RF, respectively), but much higher for FM 9058F than for ST 4554B2RF after 24 mon (9.5 × 105versus 3.8 × 10^5^ CFU/cm^3^ soil, respectively). Fusarium wilt incidence for the first, second, and third growing seasons averaged 17.9%, 33.9%, and 69.0% for FM 9058F, and 5.6%, 5.9%, and 4.3% for ST 4554B2RF, respectively. Thus, cotton cultivars can differ both in susceptibility (stand loss, vascular discoloration, and yield loss), and in ability of Fov to reproduce and buildup in the soil. The percentage mortality for all other cultivars tested in 2004 and 2005, while lower than the conventional FiberMax cultivars, were still very high (average >50% mortality) but decreased rapidly in that field when the producer switched to cultivars other than conventional FiberMax for several years (T. Wheeler, personal observations from 4 yr of cultivar trials at that site).

**Figure 3:**
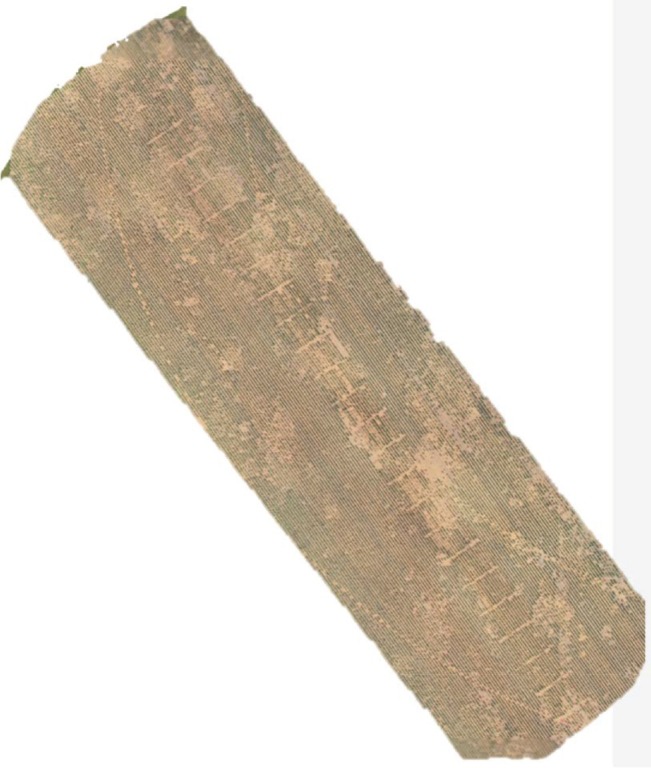
Aerial image taken in August 2019 of the test area (16 rows wide) in Gaines County showing spatial variability in stand loss due to Fusarium wilt/Mi complex. Mi, *Meloidogyne incognita*.

The catastrophic Fusarium wilt problems observed by several producers after the introduction of some *M. incognita* resistant cultivars in more recent years, was the catalyst for the 2019 to 2021 cultivar trials, and included the exact site in Gaines County where one report occurred. The Gaines County site did have the highest overall Fusarium wilt percentage mortality of all the tested sites (2019–2021); however, there were no statistical differences in mortality between the cultivar groups, indicating no high Fov resistance, even in ST 4946GLB2. This is contrasted with the significant group differences found in all tests with regard to transformed Mi density. The difficulty in testing cultivars for Fusarium wilt mortality in the field is shown in Figure 3 at the Gaines County test site. The spatial variability of the disease, even when Fusarium wilt is relatively severe, resulted in mostly non-significant differences for percentage mortality. The Hall site did have significant mortality differences between groups, but there were also 3 yr of data that could be combined at that site.

The R-DP cultivars possessed more susceptibility to Fusarium wilt than did ST 4946GLB2, R-PHY, R-FM and Mi-susceptible cultivars, even though R-DP cultivars had excellent Mi resistance. ST 4946GLB2, which was thought to be the most Fusarium wilt tolerant cultivar at the start of the 2019 to 2021 trials, did indeed have the lowest percentage mortality and highest overall lint yields in Fusarium wilt/Mi trials, but was not statistically superior to other groups except for R-DP for Fusarium wilt mortality. With regard to Mi resistance, significant separations could be seen between groups, particularly for the R-DP and R-PHY groups compared with more Mi-susceptible groups.

The original source of resistance for Mi in many cotton breeding programs was Auburn 623RNR, which was released by Shepherd (1974). This line was the most Mi and Fov resistant line available at that time in *G. hirsutum* (Shepherd, 1974). Its resistance to both these organisms greatly surpassed the resistance of Auburn 56, which until then was considered one of the most Fov and Mi-resistant varieties. There are two genes (located on chromosome 11 and 14) associated with this high Mi resistance. Gaudin and Wubben (2021) screened *G. hirsutum* accessions for resistance to Mi, and while there was a range of resistant phenotypes, the genotypic analyses revealed that all resistant accessions carried either the chromosome 11 (RK1) and/or chromosome 14 (RK2) resistance QTL. It is suspected that the high levels of resistance found in some cultivars (PHY 480W3FE, DP 2141NR B3XF, and DP 2143NR B3XF, as examples) have both Mi resistance genes, homogeneously, but these cultivars do not currently have PVP certificates available. Two other cultivars from Phytogen used in these trials were PHY 332 W3FE (PVP 202000220) and PHY 443 W3FE (PVP 202000221), and molecular markers confirmed they both have the RK1 and RK2 genes homogeneously (based on their PVP certificates). The Mi-resistant gene(s?) in partially resistant ST 4946GLB2 and R-FM group are also presumed as RK1 or RK2 genes (presumed from the work of Gaudin and Wubben [2021]) but may be present heterogeneously given the higher densities of transformed Mi found in the Fov/ Mi trials.

Mi-resistant gene(s) do not by themselves confer resistance to Fov, or there are Mi-susceptible cultivars that may have resistance to Fov (Hyer et al., 1979; Wang et al., 2009; Ulloa et al., 2011). The resistance to Fov observed in Auburn 623RNR was not simply a product of having both the chromosome 11 and chromosome 14 Mi-resistant genes, since this cultivar exhibited high resistance to both Mi and Fov (Shepherd, 1974). Marker-assisted selections have been useful for development of Mi-resistant cotton varieties. However, there is no indication within the development of U.S. cotton varieties (based on PVPs) that molecular markers are being utilized to identify Fov resistance. There have been several studies to determine the genes involved with Fov race 1 resistance in *G. hirsutum* and potential location of molecular markers (Wang et al., 2009; Ulloa et al., 2011). However, it is important that Fov (race 1 and 2) resistance genes function in the presence of Mi, since these races cause minimal losses in the absence of Mi. Development of markers for Fov (race 1 and 2) resistance and combined utilization of Mi-resistant and Fov-resistant markers would accelerate the development of Fusarium wilt resistant cultivars (to races 1 and 2).

## Figures and Tables

**Appendix 1:** Varieties used in trials.

2004	2005	2019	2019	2019	2020	2020	2021
Dawson	Dawson	Gaines	Hall	Lynn	Cochran	Hall	Hall
AFD 2428	AFD 3602 RR	Armor 9178 B3XF	Armor 9210 B3XF	DP 1522 B2XF	Armor 9210 B3XF	DP 1646 B2XF	Armor 9831 B3XF
AFD 2485	All-Tex Atlas	Armor 9210 B3XF	Armor 9598 B3XF	DP 1612 B2XF	Armor 9608 B3XF	DP 1747NR B2XF	DP 1522 B2XF
AFD 3511 RR	Americot 1621	Armor 9598 B3XF	DP 1522 B2XF	DP 1747NR B2XF	DP 1646 B2XF	DP 1840 B3XF	DP 1646 B2XF
All-Tex Atlas	Americot 8120	Armor 9608 B3XF	DP 1646 B2XF	DP 1822 XF	DP 1747NR B2XF	FM 1621GL	DP 1747NR B2XF
All-Tex Atlas RR	BCG 245	Armor 9830 B3XF	DP 1747NR B2XF	DP 1840 B3XF	DP 1820 B3XF	FM 1730GLTP	DP 1840 B3XF
All-Tex Excess RR	BCG 28R	DP 1522 B2XF	DP 1820 B3XF	DP 1909 XF	DP 1822 XF	FM 1911GLT	DP 2044 B3XF
All-Tex TopPick	BCG 295	DP 1646 B2XF	DP 1822 XF	FM 1621GL	DP 1823NR B2XF	PHY 332W3FE	DP 2141NR B3XF
All-Tex Xpress	DP 393	DP 1747NR B2XF	DP 1823NR B2XF	FM 2398GLTP	DP 1840 B3XF	PHY 400 W3FE	DP 2143NR B3XF
BCG 24R	DP 424 BGII/ RR	DP 1820 B3XF	DP 1840 B3XF	FM 2498GLT	DP 1845 B3XF	PHY 411 W3FE	FM 1730GLTP
BCG 28R	DP 434 RR	DP 1823NR B2XF	DP 1845 B3XF	FM 2574GLT	DP 1916 B3XF	PHY 443 W3FE	FM 2498GLT
BCG 30R	DP 449 BG/RR	DP 1835 B3XF	DP 1908 B3XF	NG 3930 B3XF	DP 2012 B3XF	PHY 480 W3FE	NG 4098 B3XF
DP 5415RR	DP 455 BG/RR	DP 1845 B3XF	DP 1916 B3XF	NG 4098 B3XF	DP 2020 B3XF	PHY 500 W3FE	NG 4936 B3XF
DP 555 BG/ RR	DP 491	DP 1851 B3XF	DP 1948 B3XF	PHY 400 W3FE	DP 2022 B3XF	PHY 545 W3FE	NG 5150 B3XF
FM 819	DP 493	DP 1916 B3XF	FM 1320GL	PHY 480 W3FE	DP 2038 B3XF	PHY 580 W3FE	PHY 332 W3FE
FM 832	DP 5415 RR	DP 1948 B3XF	FM 1621GL	ST 4550GLTP	DP 2044 B3XF	ST 4480B3XF	PHY 350 W3FE
FM 958	DP 555BG/RR	NG 3780 B2XF	FM 1911GLT	ST 4946GLB2	FM 1320GL	ST 4946GLB2	PHY 400 W3FE
FM 960BR	FM 958	NG 3956 B3XF	FM 2202GL	ST 5600B2XF	FM 1621 GL	ST 4990B3Xf	PHY 411 W3FE
FM 966	FM 960B2R	NG 3994 B3XF	FM 2322GL	ST 5707B2XF	FM 1730GLTP	ST 5600B2XF	PHY 443 W3FE
FM 989BR	FM 981LL	NG 4545 B2XF	FM 2398GLTP		FM 1911GLT	ST 5610B3XF	PHY 480 W3FE
PM 2167 RR	FM 989RR	NG 4777 B2XF	FM 2498GLT	DP is Deltapine	FM 2202GL		PHY 545 W3FE
PM 2280 BG/ RR	FM960RR	NG 4792 XF	FM 2574GLT	FM is FiberMax	FM 2322GL	DP is Deltapine	ST 4946GLB2
PM 2326 RR	FM966LL	ST 4946GLB2	NG 2982 B3XF	NG is NexGen	FM 2334GLT	FM is FiberMax	ST 5091B3XF
PM 2379 RR	NG 2448 R	ST 5600B2XF	NG 3500 XF	PHY is Phytogen	FM 2398GLTP	PHY is Phytogen	ST 5600B2XF
ST 2454R	NG 3969 R	ST 5707B2XF	NG 4545 B2XF	ST is Stoneville	FM 2498GLT	ST is Stoneville	ST 5707B2XF
ST 3539BR	PM 2266 RR		NG 4689 B2XF		NG 2982 B3XF		
ST 4793R	PM 2280 BG/ RR	DP is Deltapine	NG 4777 B2XF		NG 3500 XF		DP is Deltapine
ST 4892BR	PM 2379 RR	NG is NexGen	NG 4936 B3XF		NG 3956 B3XF		FM is FiberMax
ST 5599BR	ST 4686R	ST is Stoneville	PHY 210 W3FE		NG 4050 XF		PHY is Phytogen
ST LA887	ST 5303R		PHY 250 W3FE		NG 4098 B3XF		ST is Stoneville
Tamcot Sphinx	ST 5599BR		PHY 320 W3FE		NG 4689 B2XF		
			PHY 332 W3FE		NG 4792 XF		
AFD is Associated Farmers Delinting	AFD is Associated Delinting	Farmers	PHY 400 W3FE		NG 4936 B3XF		
BCG is Beltwide Cotton Genetics	BCG is Beltwide Cotton Genetics		PHY 443 W3FE		PHY 350 W3FE		
DP is Deltapine	DP is Deltapine		PHY 480 W3FE		PHY 400 W3FE		
FM is FiberMax	FM is FiberMax		PHY 500 W3FE		ST 4480 B3XF		
PM is Paymaster	NG is NexGen		PHY 545 W3FE		ST 4550GLTP		
ST is Stoneville	PM is Paymaster		PHY 580 W3FE		ST 4946GLB2		
	ST is Stoneville		ST 4550GLTP		ST 4990 B3XF		
			ST 4946GLB2		ST 5600 B2XF		
